# Influence of comorbidities on outcome in 1102 patients with an allogeneic hematopoietic stem cell transplantation

**DOI:** 10.1038/s41409-024-02395-z

**Published:** 2024-08-13

**Authors:** Marie Janscak, Anne Stelmes, Jana van den Berg, Dominik Heim, Joerg Halter, Beatrice Drexler, Christian Arranto, Jakob Passweg, Michael Medinger

**Affiliations:** 1 Division of Hematology, University Hospital Basel, Basel, Switzerland; 2 University of Basel, Basel, Switzerland

**Keywords:** Translational research, Acute myeloid leukaemia, Myelodysplastic syndrome

## Abstract

The hematopoietic comorbidity risk index (HCT-CI) is a pre-transplant risk assessment tool used to qualify comorbidities to predict non-relapse mortality (NRM) of patients undergoing allogeneic hematopoietic stem cell transplantation (allo-HSCT). HSCT procedures continue to improve. Therefore, the predictive value of HCT-CI needs to be re-evaluated. Our study is a retrospective analysis of pre-existing comorbidities assessing the relevance of the HCT-CI on the outcome of consecutive patients (*n* = 1102) undergoing allo-HSCT from 2006-2021. HCT-CI was classified as low (HCT-CI 0), intermediate (HCT-CI 1–2) and high-risk (HCT-CI ≥ 3). At 10 years, NRM for low, intermediate, and high-risk HCT-CI group was 21.0%, 26.0%, and 25.8% (*p* = 0.04). NRM difference was significant between low to intermediate (*p* < 0.001), but not between intermediate to high-risk HCT-CI (*p* = 0.22). Overall survival (OS) at 10 years differed significantly with 49.9%, 39.8%, and 31.1%, respectively (p < 0.001). In multivariate analysis of HCT-CI organ subgroups, cardiac disease was most strongly associated with NRM (HR = 1.73, *p* = 0.02) and OS (HR = 1.77, *p* < 0.001). All other individual organ comorbidities influenced NRM to a lesser extent. Further, donor (HR = 2.20, *p* < 0.001 for unrelated and HR = 2.17, *p* = 0.004 for mismatched related donor), disease status (HR = 1.41, *p* = 0.03 for advanced disease) and previous HSCT (HR = 1.55, *p* = 0.009) were associated with NRM. Improvement in transplant techniques and supportive care may have improved outcome with respect to comorbidities.

## Introduction

Allogeneic hematopoietic stem cell transplantation (allo-HSCT) is a potentially curative therapy for malignant and non-malignant hematological diseases. More patients are receiving transplants and the proportion of allografts performed in the elderly has increased recently [1]. This is mainly due to better supportive care and the use of non-myeloablative/reduced-intensity therapy schemes [2]. Because the number of pre-existing conditions increases with age, there are more comorbidities that can affect transplant-related mortality. Since the number of older patients receiving HSCT has increased, it is important to investigate the relevance of comorbidities.

The ability to predict outcome after HSCT based on pre-transplant comorbidities represents a great challenge in daily practice. Patient selection plays a central role in successful transplantation. Several scores have been described. Best known is the Hematopoietic cell transplantation comorbidity index (HCT-CI), developed and validated by Sorror et al. [3]. The HCT-CI is a pre-transplant risk assessment tool used to qualify pre-existing comorbidities to predict post-transplantation outcome, mainly non-relapse mortality (NRM) of patients undergoing allogeneic HSCT. However, the HCT-CI was originally calculated from comorbidity and NRM data of patients undergoing allo-HCT between 1997 and 2003. Since then, the predictive power of the scores for overall survival (OS) and NRM has been assessed in multiple studies. The ability to predict NRM and OS varied between studies and was not confirmed in all. Some studies showed association between HCT-CI and the OS [4–10], others failed to predict NRM and OS [11–13]. In a recently published study by Penack et al. the predictive value of the HCT-CI was estimated to be lower than previously assumed [14]. While moderate/severe renal comorbidities were strongly associated with NRM, remaining comorbidities show weaker associations. A recently developed Simplified Comorbidity Index puts stronger emphasis on renal comorbidities by including an eGFR of 60–89.9 and ≥90 mL/min per 1.73 m^2^ in the calculation among other comorbidities and was the strongest predictor of NRM and OS in a multivariable model [15].

Since donor selection, conditioning regimens and the management of post-transplant complications are improving and impact of HCT-CI is contradictory, the evaluation of comorbidities is becoming increasingly important to assess the benefits and risks of transplantation more precisely. Thus, the predictive value of HCT-CI needs to be re-evaluated. Therefore, the aim of this study is to investigate the influence of comorbidities in the HCT-CI on the outcome after allo-HSCT in a recent transplant cohort.

## Patients and methods

### Patient population and study design

Our study is a retrospective single-center analysis using electronic medical record data and was performed according to the regulations of the local ethics committee (Ethikkommission Nordwest- und Zentralschweiz Basel; study number EKNZ 2021-02397). All methods were performed in accordance with the relevant guidelines and regulations. Patients who underwent allo-HSCT between 2006 and 2021 at the University Hospital Basel over 18 years of age were included. Patients without HCT-CI data were excluded (*n* = 26). In patients receiving more than one allogeneic HSCT (*n* = 97) the first was included. Due to small numbers cord-blood (*n* = 18) and syngeneic (*n* = 8) transplants were excluded from the analysis. A total of 1102 patients were considered in the study. Variables examined were pre-existing comorbidities as well as transplant and disease characteristics, consisting of age, sex, date of transplant, disease status at transplant, disease classification, number of previous autologous HSCT, HSCT source, donor type (sibling, unrelated, mismatch related), performance status (KPS), intensity and type of conditioning regimen (myeloablative conditioning (MAC) or reduced-intensity conditioning (RIC)), use of anti-thymocyte globulin (ATG), occurrence of acute or chronic graft-versus-host disease (GvHD) and the cause of death [16]. One antigen mismatched related donors were grouped with matched related and involves <1% of the donors. Two or more antigen mismatched related donors i.e. the vast majority of which were haploidentical were grouped with the mismatched related donors.

The outcomes of interest were NRM, OS, progression-free survival (PFS), relapse incidence (RI), incidence of acute and chronic GvHD and cause of death.

### Conditioning regimens and GvHD prophylaxis

Cyclophosphamide combined with busulfan, cyclophosphamide and total body irradiation (TBI) ≥ 8 Gy, cytarabine, carmustine, etoposide, melphalan and fludarabine (BEAM- fludarabine) and further protocols were used in MAC regimens [17]. RIC regimens included fludarabine with low-dose TBI < 6 Gy, fludarabine combined with busulfan or melphalan and other protocols. Older age or relevant comorbidities were reasons for RIC.

Within MAC conditioning regimens, GvHD prophylaxis was with cyclosporine A (CsA) and methotrexate (MTX). According to institutional standards, if RIC was fludarabine/busulfan, GvHD prophylaxis consisted of CsA and MTX or CsA and mycophenolate mofetil (MMF) in cases of RIC with fludarabine/low-dose TBI. In haploidentical donors the GvHD prophylaxis consisted of post-transplant cyclophosphamide (PTCy), CsA and MMF. In case the donor was unrelated or matched but donor or recipient were ≥ 40 years old, ATG was used [18].

Clinical symptoms and biopsies were used to diagnose acute and chronic GvHD, which were graded according to consensus criteria [19, 20].

### Assessment of comorbidities

Comorbidities were assessed using electronic medical record data and the HCT-CI index was calculated according to Sorror et al. [3]. HCT-CI was classified as low risk (HCT-CI 0), intermediate risk (HCT-CI 1–2) and high risk (HCT-CI ≥ 3).

### Statistical analysis

The primary study endpoint was to assess the prevalence of pretransplant comorbidities according to the HCT-CI and impact on NRM. NRM is defined as death without malignancy. Secondary study endpoints were OS, PFS, RI, incidence of acute and chronic GvHD and cause of death. For all endpoints, start time was the transplant date. Continuous variables are presented as median (range) and categorial variables as absolute values and percentages. Patient and disease characteristics are evaluated using descriptive statistics. Chi-square tests are used to determine significant differences in the distribution of parameters.

Cumulative incidence functions were used to estimate NRM and RI considering NRM or relapse as the respective competing risks. Time-to-event outcome for acute GvHD (grade II+) and chronic GvHD (any extent) were estimated using cumulative incidence curves, using death and relapse as competing risks. Differences among groups were compared using Gray’s test. OS and PFS were calculated using the Kaplan-Meier estimator. Comparisons were by the log-rank test. OS was determined form allo-HSCT until death from any cause and PFS was calculated from allo-HSCT to relapse or death.

Multivariable models for OS were performed by using a Cox model. For estimating hazard ratios of NRM the Fine-Gray competing risk model was used. Variables were included in the model using a stepwise backward elimination with a threshold *p* < 0.05. Each comorbidity was included individually in a model adjusting for significant non-HCT-CI covariates (NRM: age, disease stage, donor, presence of a previous transplantation and CMV. OS: age, disease classification, disease stage, performance status and CMV). Results were expressed as hazard ratio (HR) with 95% confidence interval (CI) shown as forest plots. A *p* value of <0.05 is considered statistically significant. Statistical analyses were performed with SPSS (version 28; IBM, Chicago, IL, USA) and STATA (version 18; StataCorp LLC, College Station, TX, USA) software.

## Results

### Patient characteristics

Between January 2006 and November 2021, we identified 1102 patients who received an allo-HSCT at the University Hospital Basel fulfilling the inclusion criteria. HSCT was used to treat AML (*n* = 423, 38.4%), MDS/MPN (*n* = 247, 22.4%), CML/CLL (*n* = 71, 6.4%), ALL (*n* = 133, 12.1%), Lymphoma/Myeloma (*n* = 185, 16.8%) and others (*n* = 43, 3.9%). The median age was 54.6 years, ranging from 18.2 to 75.8. MAC was used in 735 (66.7%) and RIC in 364 (33.0%). Patient characteristics are shown in Table 1.

**Table 1:** Patient characteristics.

Baseline characteristics	Overall (*n* = 1102)	HCT-CI = 0 (*n* = 554)	HCT-CI = 1 or 2 (*n* = 303)	HCT-CI = ≥ 3 (*n* = 245)	*p*-value
Disease classification					
AML & related precursor neoplasms	423 (38.4%)	200 (36.1%)	131 (43.2%)	92 (37.6%)	*p* = 0.001
Precursor lymphoid neoplasms	133 (12.1%)	76 (13.7%)	34 (11.2%)	23 (9.4%)	
CML / CLL	71 (6.4%)	44 (7.9%)	16 (5.3%)	11 (4.5%)	
Lymphoma or Plasma cell disorders	185 (16.8%)	111 (20.0%)	41 (13.9%)	32 (13.1%)	
MDS/MPN	247 (22.4%)	107 (19.3%)	69 (22.8%)	71 (29.0%)	
Bone marrow failure or others	43 (3.9%)	16 (2.9%)	11 (3.6%)	16 (6.5%)	
Disease status at transplant					
Complete remission (1st or 2nd)	614 (55.8%)	338 (61.0%)	153 (50.7%)	123 (50.2%)	*p* < 0.001
Advanced disease	428 (38.9%)	188 (33.9%)	139 (46.0%)	101 (41.2%)	
Missing	59 (5.4%)	28 (5.1%)	10 (3.3%)	21 (8.6%)	
Cell source					
Peripheral blood	981 (89.0%)	500 (90.3%)	266 (87.8%)	215 (87.8%)	*p* = 0.42
Bone marrow	121 (11.0%)	54 (9.7%)	37 (12.2%)	30 (12.2%)	
Patient sex					
Female	415 (37.7%)	205 (37.0%)	117 (38.6%)	93 (38.0%)	*p* = 0.89
Male	687 (62.3%)	349 (63.0%)	186 (61.4%)	152 (62.0%)	
Patient age					
Median (min-max) [IQR]	54.6 (18.2–75.8)	51.2 (18.2–75.8)	55.2 (18.7–75.7)	58.3 (18–73.5)	*p* < 0.001
Age category					
18–20 years	17 (2.0%)	11 (1.0%)	3 (0.3%)	3 (0.3%)	*p* < 0.001
20–30 years	92 (8.3%)	56 (10.1%)	23 (7.6%)	13 (5.3%)	
30–40 years	123 (11.2%)	71 (12.8%)	30 (9.9%)	22 (9.0%)	
40–50 years	200 (18.1%)	117 (21.1%)	57 (18.8%)	26 (10.6%)	
50–60 years	305 (27.7%)	143 (25.8%)	92 (30.4%)	70 (28.6%)	
60–70 years	316 (28.7%)	135 (24.4%)	86 (28.4%)	95 (38.8%)	
Over 70 years	49 (4.4%)	21 (3.8%)	12 (4.0%)	16 (6.5%)	
Donor type					
Sibling	388 (35.2%)	211 (38.1%)	99 (32.7%)	78 (31.8%)	*p* = 0.03
Unrelated	620 (56.3%)	308 (55.6%)	172 (56.8%)	140 (57.1%)	
Mismatched related	94 (8.5%)	35 (6.3%)	32 (10.6%)	27 (11.0%)	
Cytomegalovirus (CMV) patient					
Positive	631 (57.3%)	298 (53.8%)	188 (62.0%)	145 (59.2%)	*p* = 0.05
Negative	471 (42.7%)	256 (46.2%)	115 (38.0%)	100 (40.8%)	
Previous transplant					
Yes	217 (19.7%)	123 (22.2%)	60 (19.8%)	34 (13.9%)	*p* = 0.02
No	885 (80.3%)	431 (77.8%)	243 (80.2%)	211 (86.1%)	
Performance status					
Over 90%, good	846 (76.8%)	472 (85.2%)	224 (73.9%)	150 (61.2%)	*p* < 0.001
Under 90%, poor	251 (22.8%)	81 (14.6%)	79 (26.1%)	91 (37.1%)	
Missing	5 (0.5%)	1 (0.2%)	0 (0.0%)	4 (1.6%)	
Intensity of conditioning					
Myeloablative (MAC)	735 (66.7%)	406 (73.3%)	196 (64.7%)	133 (54.3%)	p < 0.001
Reduced intensity (RIC)	364 (33.0%)	147 (26.5%)	106 (35.0%)	111 (45.3%)	
Missing	3 (0.3%)	1 (0.2%)	1 (0.3%)	1 (0.4%)	
Conditioning regimen					
BuCy	274 (24.9%)	159 (28.7%)	76 (25.2%)	39 (15.9%)	*p* < 0.001
FluBu	294 (26.7%)	113 (20.4%)	94 (31.1%)	87 (35.5%)	
CyTBI	138 (12.5%)	91 (16.4%)	29 (9.6%)	18 (7.3%)	
FluTBI mini	186 (16.9%)	85 (15.3%)	56 (18.5%)	45 (18.4%)	
Myeloablative other	116 (10.5%)	68 (12.3%)	21 (7.0%)	27 (11.0%)	
Reduced intensity other	51 (4.6%)	21 (3.8%)	11 (3.6%)	19 (7.8%)	
TBI other	42 (3.8%)	17 (3.1%)	15 (5.0%)	10 (4.1%)	
Anti-thymocyte globulin (ATG)					
Yes	383 (34.8%)	188 (33.9%)	104 (34.3%)	91 (37.3%)	*p* = 0.64
No	718 (65.2%)	366 (66.1%)	199 (65.7%)	153 (62.7%)	

*AML* acute myeloid leukemia*, CML* chronic myeloid leukemia*, CLL* chronic lymphatic leukemia*, MDS* myelodysplastic syndrome*, MPN* myeloproliferative neoplasm*, min* minimum*, max* maximum*, Bu* busulfan*, Cy cyclophosphamide, Flu* fludarabine*, TBI* total body irradiation.

### Prevalence of pre-transplant comorbidities

**Fig. 1:**
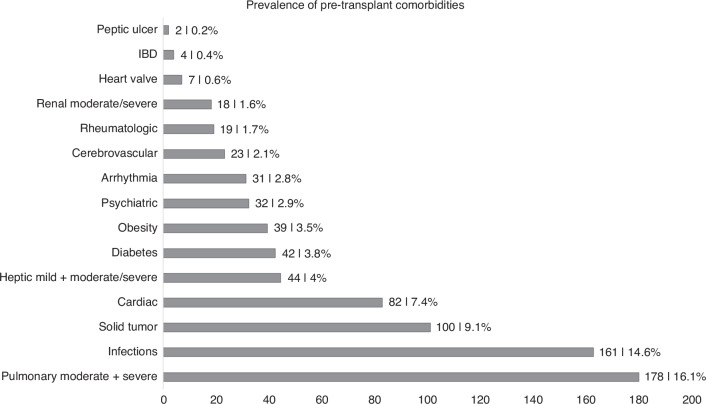
Prevalence of pre-transplant comorbidities (*n* = 1102).

The prevalence of pre-transplant comorbidities was investigated. Pulmonary comorbidities were most frequent (16.1%) in the study population, followed by infections (14.6%), solid tumor (9.1%) and cardiac comorbidity (7.4%). More rarely hepatic comorbidities (4.0%), diabetes (3.8%), obesity (3.5%) and psychiatric disease (2.9%) were found. Less often there were arrhythmias (2.8%), cerebrovascular disease (2.1%), rheumatologic comorbidity (1.7%), renal comorbidity (1.6%), heart valve (0.6%), inflammatory bowel disease (0.4%) and peptic ulcer (0.2%) (Fig. 1).

Five hundred fifty-four patients (50.3%) had an HCT-CI = 0 (low risk, no comorbidities). Three hundred and three (27.5%) had an HCT-CI = 1–2 (intermediate risk) and 245 patients (22.2%) had an HCT-CI ≥ 3 (high-risk).

### Outcomes

**Fig. 2:**
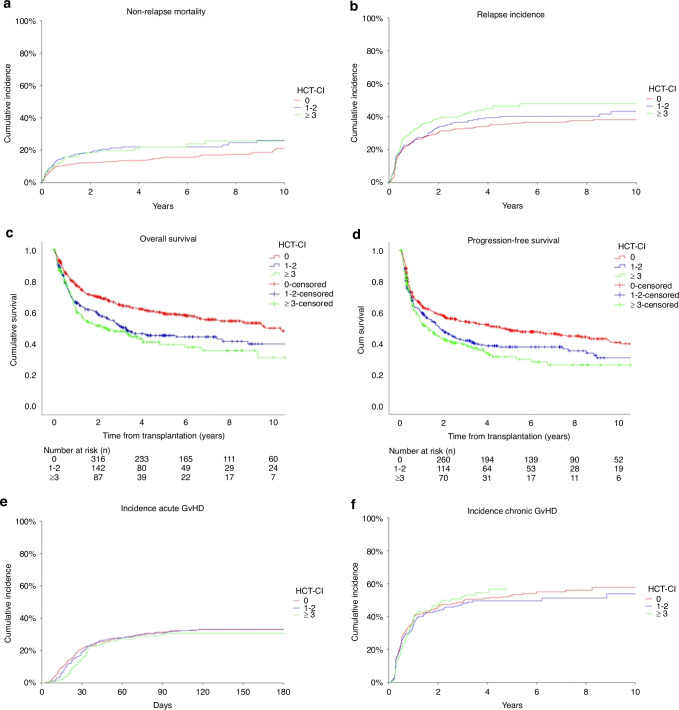
Association of outcome variables with HCT-CI. **a** Non-relapse mortality. **b** Relapse incidence. **c** Overall survival. **d** Progression-free survival. **e** Incidence of acute GvHD. **f** Incidence of chronic GvHD.

We investigated the association between HCT-CI with NRM, OS, and PFS. NRM at 10 years for low, intermediate, and high-risk HCT-CI differed significantly with 21.0%, 26.0%, and 25.8% (*p* = 0.04). The overall survival at 10 years was 49.9%, 39.8%, and 31.1%, respectively (*p* < 0.001). At 10 years the PFS shows a significant difference between the HCT-CI groups with 40.7%, 30.8%, and 26.2% (*p* < 0.001). The relapse incidence was at 10 years 38.2%, 43.2%, 47.9%, of borderline significance (*p* = 0.07). The incidence of acute GvHD grade II–IV at 100 days was 32.4%, 32.0%, and 30.5% and of the chronic GvHD at 2 years was 45.5%, 43.5%, and 47.3% and showed no significant difference (Fig. 2; Table 2).

**Table 2:** Outcome variables.

Outcome variable	Time	HCT-CI = 0 (*n* = 554)	HCT-CI = 1 or 2 (*n* = 303)	HCT-CI = ≥ 3 (*n* = 245)	*p* value
Non-relapse mortality	At 1 year	10.9% (8.5–13.9)	15.5% (11.8–20.4)	15.5% (11.3–21.3)	*p* = 0.04
	At 2 years	12.1% (9.6–15.3)	18.8% (14.7–24.0)	18.4% (13.8–24.6)	
	At 5 years	15.4% (12.4–19.0)	22.0% (17.5–27.7)	22.1% (16.6–29.3)	
	At 10 years	21.0% (16.9–26.2)	26.0% (20.3–33.3)	25.8% (19.1–34.7)	
Overall survival	At 1 year	77.5% ± 4	66.4% ± 6	62.0% ± 7	*p* < 0.001
	At 2 years	69.8% ± 4	59.2% ± 6	51.4% ± 7	
	At 5 years	59.3% ± 5	45.2% ± 7	39.3% ± 8	
	At 10 years	49.9% ± 6	39.8% ± 8	31.1% ± 12	
Progression-free survival	At 1 year	63.5% ± 4	59.5% ± 6	52.2% ± 7	*p* < 0.001
	At 2 years	57.5% ± 4	47.7% ± 6	42.5% ± 7	
	At 5 years	48.6% ± 5	37.9% ± 6	31.5% ± 8	
	At 10 years	40.7% ± 6	30.8% ± 8	26.2% ± 9	
Relapse incidence	At 1 year	25.6% (22.1–29.7)	25.0% (20.4–30.6)	32.1% (26.3–39.2)	*p* = 0.07
	At 2 years	30.3% (26.6–34.6)	33.5% (28.3–39.6)	39.0% (32.7–46.4)	
	At 5 years	36.0% (31.9–40.6)	40.1% (34.4–46.8)	46.4% (39.3-54.8)	
	At 10 years	38.2% (33.9–43.1)	43.2% (36.6–50.9)	47.9% (40.5-56.7)	
Incidence acute GvHD	At 100 days	32.4% (28.5–36.7)	32.0% (27.0–38.0)	30.5% (25.0–37.3)	*p* = 0.66
	At 1 year	33% (29.1–37.4)	33.2% (28.1–39.2)	30.5% (25.0–37.3)	
Incidence chronic GvHD	At 1 year	39.0% (34.8–43.7)	34.7% (29.2–41.3)	39.1% (32.5–47.0)	*p* = 0.71
	At 2 years	45.5% (41.1-50.4)	43.5% (37.6–50.4)	47.3% (40.4–55.5)	

*GvHD* graft-versus-host disease.

### Cause of death

At follow-up 475 (43.1%) patients had died. The most common cause of death was relapse/progression of the original disease (57.3%). Out of all deaths 187 patients (39.4%) died due to NRM. Most of the transplant-related mortality was infection related (9.3%), followed by GvHD (9.1%), other causes (8.6%) and organ failure (6.5%). There was no significant difference among the HCT-CI groups (Table 3).

**Table 3:** Cause of death.

Cause of death	Overall (*n* = 475)	HCT-CI = 0 (*n* = 215)	HCT-CI = 1 or 2 (*n* = 145)	HCT-CI = ≥ 3 (*n* = 115)	*p* value
Disease-related mortality (DRM)					*p* = 0.80
Relapse or progression of original disease	272 (57.3%)	124 (57.7%)	80 (55.2%)	68 (59.1%)	
Transplant-related mortality (TRM)					
Infection related	44 (9.3%)	19 (8.8%)	15 (10.3%)	10 (8.7%)	
Infection related + GvHD	21 (4.4%)	10 (4.7%)	10 (6.9%)	1 (0.9%)	
GvHD	43 (9.1%)	23 (10.7%)	11 (7.6%)	9 (7.8%)	
Organ failure	31 (6.5%)	15 (7.0%)	8 (5.5%)	8 (7.0%)	
Organ failure + Infection related	7 (1.5%)	4 (1.9%)	1 (0.7%)	2 (1.7%)	
Other^a^	41 (8.6%)	16 (7.4%)	13 (9.0%)	12 (10.4%)	
Missing	5 (1.1%)	2 (0.9%)	2 (1.4%)	1 (0.9%)	

^a^Other = other cause of death in the absence of disease progression/relapse, GvHD (graft versus host disease), infection or organ failure, including primary or secondary graft failure, thrombotic or hemorrhagic events, second malignancies, unknown.

*GvHD* graft-versus-host disease.

### Multivariate analysis

#### HCT-CI

**Fig. 3:**
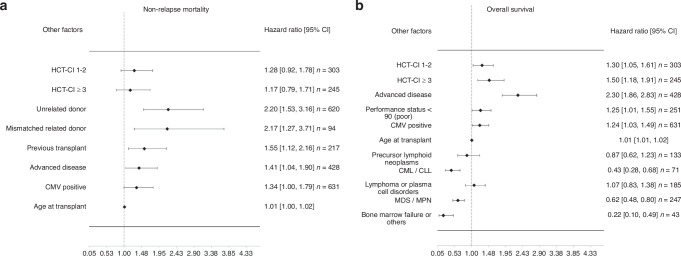
Association of other transplant-relevant factors with outcome variables. **a** Non-relapse mortality. **b** Overall survival.

By multivariate analysis, the HR for NRM were 1.28 (95% CI 0.92–1.78, *p* = 0.15) in patients with HCT-CI 1–2 and 1.17 (95% CI 0.79–1.71, *p* = 0.44) in patients with HCT-CI ≥ 3; the risk for NRM was not significantly increased (Fig. 3a). The respective HR for OS were 1.30 (95% CI 1.05–1.61, *p* = 0.018) in patients with HCT-CI 1–2 and 1.50 (95% CI 1.18–1.91, *p* = 0.001) in patients with HCT-CI ≥ 3 and a significant increase in risk for OS was shown (Fig. 3b).

#### Individual comorbidities

**Fig. 4:**
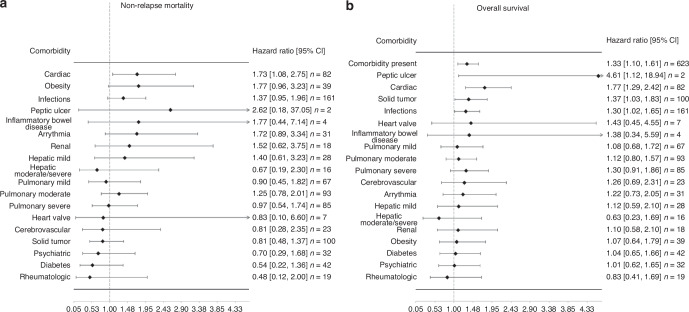
Association of individual comorbidities with outcome variables. **a** Non-relapse mortality. **b** Overall survival.

In multivariate analysis, regarding the association of individual organ pre-transplant comorbidities with NRM we found a strong significant association with cardiac disease (HR 1.73, 95% CI 1.08–2.75, *p* = 0.02) with NRM (Fig. 4a). Regarding overall survival, cardiac disease was most strongly associated (HR 1.77, 95% CI 1.29–2.42, *p* < 0.001). Furthermore, solid tumors, infections and peptic ulcer are significantly associated with OS with a HR 1.37 (95% CI 1.03–1.83, *p* = 0.03),HR 1.30 (95% CI 1.02–1.65, *p* = 0.03) and HR 4.61 (95% CI 1.12–18.94, respectively (Fig. 4b). Other individual organ comorbidities were not significantly associated with outcomes.

#### Other significant covariates

Disease stage (HR 1.41, 95% CI 1.04-1.90, *p* = 0.026 for advanced disease), prior autologous HSCT (HR 1.55, 90% CI 1.12–2.16, *p* = 0.009), donor (HR 2.20, 95% CI 1.53-3.16, *p* < 0.001 for unrelated and HR 2.17, 95% CI 1.27–3.71, *p* = 0.004 for mismatched related), CMV state (HR 1.34, 95% CI 1.00–1.79, *p* = 0.05 for CMV positive patients) and age (HR 1.01, 95% CI 1.00–1.02, *p* = 0.019) were also significantly associated with NRM (Fig. 3a).

Regarding OS, there was a significant association with performance status (HR 1.25, 95% CI 1.01–1.55 *p* = 0.037 for performance status below 90%), disease status (HR 2.30, 95% CI 1.86–2.83, *p* < 0.001, for advanced disease), CMV state (HR 1.24, 95% CI 1.03–1.49, *p* = 0.026 for CMV positive patients) and age (HR 1.01, 95% CI 1.01–1.02, *p* = 0.001). There was a significance in disease classification in CML/CLL (HR 0.43, 95% CI 0.28–0.68, p < 0.001), MDS/MPN (HR 0.62, 95% CI 0.48–0.80, *p* < 0.001) and bone marrow failure and others (HR 0.22, 95% CI 0.10–0.49, *p* < 0.001) in comparison with AML, regarding OS (Fig. 3b).

### Correlation of comorbidities

Solid tumor and heart valve disease show no correlation to other comorbidities. There were significant correlations between comorbidities, in particular IBD with infections, diabetes and obesity, rheumatologic disorders with peptic ulcer disease, diabetes with hepatic, cardiac, pulmonary complications and obesity, renal with hepatic and hepatic with pulmonary disease and obesity, arrhythmia with pulmonary complications and cardiac with cerebrovascular; all these correlations were somehow to be expected based on general internal medicine pathophysiology, the correlation of infection with psychiatric disorders is weak and most likely of no clinical relevance. Further, there are only few patients with IBD, pulmonary and heart valve disease, so these correlations are difficult to interpret. We nevertheless treated individual comorbidities as single entities as shown in Fig. 4 analyzing impact on NRM and survival. A correlation matrix of individual comorbidities showing this in more detail is shown (Supplementary Fig. 1), where the bright red or blush red color indicates a stronger or weaker correlation among 2 variables.

## Discussion

The HCT-CI is a pre-transplant risk assessment tool used to qualify comorbidities to predict NRM of patients undergoing allogeneic HSCT. Since HSCT procedures continue to improve and the comorbidities increases with age the predictive value of HCT-CI in the aging population needs to be reevaluated. In the current analysis, we examined a large representative data set consisting of 1102 patients over a long observation period of about 15 years.

We found a high prevalence of comorbidities in allogeneic transplanted patients as shown in previously published studies [10, 14, 21]. The most common pre-transplant comorbidities were reduced pulmonary function, infections and prior solid tumor. When comparing our data with the original study by Sorror et al. although pulmonary comorbidity occurred most, the frequency is 24% Sorror vs 16.1% our study [3]. Furthermore, in the current EBMT population and comparable studies pulmonary comorbidity occurred at a similar frequency (21.4% vs our study 16.1%) followed by infections (7.1% vs 14.6%), cardiac comorbidity (5.6% vs 7.4%) and solid tumor (5.2% vs 9.1%) [14, 22, 23]. However, when looking at Sorror et al. there are some differences in the prevalence of comorbidities – however, pulmonary comorbidity occurred most frequently in both [3]. Since we used the same definitions for the HCT-CI we consider our data to be comparable and generalizable. We explain the more frequent occurrence of comorbidities by the fact that our population is on average 10 years older (54.6 years vs 44.8 years). For this reason, the score might not be fully applicable and new or adapted scores tailored to the baseline characteristics may be required.

The multivariate analysis showed that cardiac comorbidity is most strongly associated with NRM. Comparable results were obtained in the study by Khalil et al. (HR 1.73 vs HR 1.78), but diabetes mellitus had the greatest influence [9]. Comparing a retrospective study, there was a significant increased NRM risk with cardiac disease in patients conditioned with fludarabine/busulfan (HR 5.54). The conditioning regimen influences the risk associated with specific comorbidities [24]. In addition, Terwey et al. also showed that cardiac disease is predictive of an increase in NRM (HR 3.9, *p* = 0.005) [25]. However, there is a higher NRM-associated risk comparing the impact of comorbidities described by Sorror et al. The HR is 3 or higher, whereas in our study the strongest association with NRM was observed with cardiac comorbidity but with an HR just below 2 [3]. Thus, we observe a reduced effect of comorbidities on NRM compared to the original publication. Compared to a prospective study by Sorror et al examining the predictive power of the HCT-CI, NRM was higher and OS worse than in our study [26]. We believe that these differences are due to patient selection and adapted transplantation methods compared to previous periods by using less toxic conditioning regimens (RIC) [27]. We analyzed changes in conditioning regimens in the periods 2006–2014 vs. 2015–2021 and found a percentage increase in the use of RIC. From 2006–2014, 371 (76.7%) patients received MAC and only 112 (23.1%) received RIC. In the later period, MAC was used in 364 (58.9%) of patients and RIC in 252 (40.8%). There is also a better surveillance and supportive care, as well as improved management of post-transplant complications and improved infection-related mortality by using more antifungal and -viral drugs and increased use of peripheral blood transplant leading to a shorter period of neutropenia [28, 29]. Increased use of in-vivo T-cell depletion resulted in a lower risk of GvHD [18].

Patients with low, intermediate and high HCT-CI differ in many characteristics. We found a significant NRM difference between low to intermediate (*p* < 0.01), but not between intermediate and high-risk patients (*p* = 0.22). The cut off points of low, intermediate and high-risk may be a limitation of the index. Some studies have shown that a modified or flexible HCT-CI may increase its predictive power [22, 30]. Recently, the Simplified Comorbidity Index, which is composed of 4 comorbidities and age older than 60 years and stratifies patients into 5 groups, showed better discrimination of outcomes than the original HCT-CI [15].

According to our study, the relapse incidence in the HCT-CI groups showed a borderline significance of *p* = 0.07. We assume that this is largely age related with more aggressive disease and comorbidities being more prevalent with age. In addition this could be explained by the interaction of comorbidities and cancer [31]. Studies have shown that patients with comorbidities such as obesity, diabetes and chronic lung disease have a higher risk developing a hematologic malignancy [32, 33]. This was also demonstrated in other studies, which have shown that higher HCT-CI scores are associated with an increased risk of relapse [34, 35]. Moreover, comorbidities influence transplant strategy, e.g. conditioning intensity and use of post-transplantation interventions such as donor lymphocyte infusion or maintenance therapies and thus affect the relapse incidence.

When comparing HCT-CI in multivariate analysis, there is an increase in the HR of the intermediate and high-risk group of HCT-CI for NRM (HR 1.28 [0.92–1.78], HR 1.17 [0.79–1.71]) and OS (HR 1.30 [1.05–1.61], HR 1.50 [1.18–1.91]). Other covariates such as prior autologous HSCT (HR 1.55 [1.12–2.16]), advanced disease (HR 1.41 [1.04–1.90]) and unrelated (HR 2.20 [1.53–3.16]) or mismatched related donor (HR 2.17 [1.27–3.71]) also have an impact on NRM and even a greater one when looking at HR. Some previous studies combined comorbidities with other risk factors for transplantation [36]. For example, Sorror et al. showed that combining comorbidities with disease status allows for better stratification of patients at high risk of NRM [34]. In addition, others showed that disease stage is a predominant prognostic factor [25]. We have shown that patients with low HCT-CI tend to be transplanted at an earlier disease stage.

In contrast to the HCT-CI, which only contains comorbidities, the European Group for Blood and Marrow Transplantation (EBMT) -score includes transplant-related variables, among others disease stage. On this subject, Barba et al. showed that a consideration of the EBMT-score in high-risk patients (HCT-CI ≥ 3) results in a better discrimination [37]. However, there is still a lack of data how multiple scores can be integrated to better predict NRM and OS. In contrast, one study showed that HCT-CI, EBMT or PAM (Pretransplant Assessment of Mortality) do not predict NRM or OS in univariate analysis in elderly people receiving a reduced-intensity conditioning [13, 38]. Although, in a large cohort for various hematological disorders, the rPAM score was validated as an independent predictor of OS and that NRM increased with higher rPAM scores [38].

We acknowledge the limitation of a retrospective study with collection of risk score parameters and comorbidities as well as a single-center design. Moreover, the data collection lasted 15 years and thus transplantation methods and supportive care may have changed during this time. However, this allowed more patients to be included, resulting in a longer observation period with valuable data. Thus, our patient population consists of a natural heterogeneity including all age groups form 18 to >75 years. We did not distinguish between elderly population, different hematological diseases or subgroup analyses by treatment era. The unavailability of certain lab parameters precluded the assessment of more contemporary comorbidity indices such as the simplified HCT-CI.

Further studies should address not only the additive effect of each comorbidity but also the assessment of interaction of each comorbidity with patient and disease characteristics or explore the shifts in the HCT-CI from first to second allo-HSCT. In future analysis, the correlation of defined comorbidities in clusters (eg the ‘metabolic syndrome’ cluster, the cardiac/cardiovascular disease cluster); could lead to unique transplant strategies for patients sharing these sets of comorbidities. Moreover, it would be interesting to integrate multiple scores to better predict NRM and OS.

In conclusion, we found no difference regarding NRM between intermediate and high-risk HCT-CI group. Cardiac comorbidity had the strongest association with NRM. All other comorbidities influenced NRM to a much lesser extent than described in previous studies. Improvements in transplant techniques and supportive care may have improved outcome with respect to comorbidities in HSCT.

## Supplementary information

Supplementary_material

## Data Availability

The datasets generated during and/or analyzed during the current study are available from the corresponding author on reasonable request.

## References

[1] Muffly L, Pasquini MC, Martens M, Brazauskas R, Zhu X, Adekola K, et al. Increasing use of allogeneic hematopoietic cell transplantation in patients aged 70 years and older in the United States. Blood. 2017;130:1156–64. 28674027 10.1182/blood-2017-03-772368 PMC5580273

[2] Gooley TA, Chien JW, Pergam SA, Hingorani S, Sorror ML, Boeckh M, et al. Reduced mortality after allogeneic hematopoietic-cell transplantation. N Engl J Med. 2010;363:2091–101. 21105791 10.1056/NEJMoa1004383 PMC3017343

[3] Sorror ML, Maris MB, Storb R, Baron F, Sandmaier BM, Maloney DG, et al. Hematopoietic cell transplantation (HCT)-specific comorbidity index: a new tool for risk assessment before allogeneic HCT. Blood. 2005;106:2912–9. 15994282 10.1182/blood-2005-05-2004 PMC1895304

[4] Sorror M, Storer B, Sandmaier BM, Maloney DG, Chauncey TR, Langston A, et al. Hematopoietic cell transplantation-comorbidity index and Karnofsky performance status are independent predictors of morbidity and mortality after allogeneic nonmyeloablative hematopoietic cell transplantation. Cancer. 2008;112:1992–2001. 18311781 10.1002/cncr.23375

[5] Sorror ML, Giralt S, Sandmaier BM, De Lima M, Shahjahan M, Maloney DG, et al. Hematopoietic cell transplantation specific comorbidity index as an outcome predictor for patients with acute myeloid leukemia in first remission: combined FHCRC and MDACC experiences. Blood. 2007;110:4606–13. 17873123 10.1182/blood-2007-06-096966 PMC2234788

[6] El Kourashy S, Williamson T, Chaudhry MA, Savoie ML, Turner AR, Larratt L, et al. Influence of comorbidities on transplant outcomes in patients aged 50 years or more after myeloablative conditioning incorporating fludarabine, BU and ATG. Bone Marrow Transplant. 2011;46:1077–83. 21057555 10.1038/bmt.2010.257

[7] Giles FJ, Borthakur G, Ravandi F, Faderl S, Verstovsek S, Thomas D, et al. The haematopoietic cell transplantation comorbidity index score is predictive of early death and survival in patients over 60 years of age receiving induction therapy for acute myeloid leukaemia. Br J Haematol. 2007;136:624–7. 17223919 10.1111/j.1365-2141.2006.06476.x

[8] Keller JW, Andreadis C, Damon LE, Kaplan LD, Martin TG, Wolf JL, et al. Hematopoietic cell transplantation comorbidity index (HCT-CI) is predictive of adverse events and overall survival in older allogeneic transplant recipients. J Geriatr Oncol. 2014;5:238–44. 24894413 10.1016/j.jgo.2014.04.003

[9] Khalil MMI, Lipton JH, Atenafu EG, Gupta V, Kim DD, Kuruvilla J, et al. Impact of comorbidities constituting the hematopoietic cell transplant (HCT)-comorbidity index on the outcome of patients undergoing allogeneic HCT for acute myeloid leukemia. Eur J Haematol. 2018;100:198–205. 29168234 10.1111/ejh.13000

[10] Veeraputhiran M, Yang L, Sundaram V, Arai S, Lowsky R, Miklos D, et al. Validation of the hematopoietic cell transplantation-specific comorbidity index in nonmyeloablative allogeneic stem cell transplantation. Biol Blood Marrow Transplant. 2017;23:1744–8. 28668491 10.1016/j.bbmt.2017.06.005 PMC5873304

[11] Guilfoyle R, Demers A, Bredeson C, Richardson E, Rubinger M, Szwajcer D, et al. Performance status, but not the hematopoietic cell transplantation comorbidity index (HCT-CI), predicts mortality at a Canadian transplant center. Bone Marrow Transplant. 2009;43:133–9. 18762762 10.1038/bmt.2008.300

[12] Birninger N, Bornhäuser M, Schaich M, Ehninger G, Schetelig J. The hematopoietic cell transplantation-specific comorbidity index fails to predict outcomes in high-risk AML patients undergoing allogeneic transplantation–investigation of potential limitations of the index. Biol Blood Marrow Transplant. 2011;17:1822–32. 21708108 10.1016/j.bbmt.2011.06.009

[13] Castagna L, Fürst S, Marchetti N, El Cheikh J, Faucher C, Mohty M, et al. Retrospective analysis of common scoring systems and outcome in patients older than 60 years treated with reduced-intensity conditioning regimen and alloSCT. Bone Marrow Transplant. 2011;46:1000–5. 20921945 10.1038/bmt.2010.227

[14] Penack O, Peczynski C, Mohty M, Yakoub-Agha I, de la Camara R, Glass B, et al. Association of pre-existing comorbidities with outcome of allogeneic hematopoietic cell transplantation. A retrospective analysis from the EBMT. Bone Marrow Transplant. 2021;57:183–90. 34718346 10.1038/s41409-021-01502-8 PMC8821004

[15] Elias S, Brown S, Devlin SM, Barker JN, Cho C, Chung DJ, et al. The Simplified Comorbidity Index predicts non-relapse mortality in reduced-intensity conditioning allogeneic haematopoietic cell transplantation. Br J Haematol. 2023;203:840–51. 37614192 10.1111/bjh.19055 PMC10843799

[16] Hahn T, Sucheston-Campbell LE, Preus L, Zhu X, Hansen JA, Martin PJ, et al. Establishment of definitions and review process for consistent adjudication of cause-specific mortality after allogeneic unrelated-donor hematopoietic cell transplantation. Biol Blood Marrow Transplant. 2015;21:1679–86. 26028504 10.1016/j.bbmt.2015.05.019 PMC4537799

[17] Bianchi M, Heim D, Lengerke C, Halter J, Gerull S, Kleber M, et al. Cyclosporine levels > 195 μg/L on day 10 post-transplant was associated with significantly reduced acute graft-versus-host disease following allogeneic hematopoietic stem cell transplantation. Ann Hematol. 2019;98:971–7. 30542943 10.1007/s00277-018-3577-1

[18] Binkert L, Medinger M, Halter JP, Heim D, Gerull S, Holbro A, et al. Lower dose anti-thymocyte globulin for GvHD prophylaxis results in improved survival after allogeneic stem cell transplantation. Bone Marrow Transplant. 2015;50:1331–6. 26121111 10.1038/bmt.2015.148

[19] Przepiorka D, Weisdorf D, Martin P, Klingemann HG, Beatty P, Hows J, et al. 1994 Consensus conference on acute GVHD grading. Bone Marrow Transplant. 1995;15:825–8. 7581076

[20] Filipovich AH, Weisdorf D, Pavletic S, Socie G, Wingard JR, Lee SJ, et al. National Institutes of Health consensus development project on criteria for clinical trials in chronic graft-versus-host disease: I. Diagnosis and staging working group report. Biol Blood Marrow Transplant. 2005;11:945–56. 16338616 10.1016/j.bbmt.2005.09.004

[21] Versluis J, Labopin M, Niederwieser D, Socie G, Schlenk RF, Milpied N, et al. Prediction of non-relapse mortality in recipients of reduced intensity conditioning allogeneic stem cell transplantation with AML in first complete remission. Leukemia. 2015;29:51–7. 24913728 10.1038/leu.2014.164

[22] Barba P, Piñana JL, Martino R, Valcárcel D, Amorós A, Sureda A, et al. Comparison of two pretransplant predictive models and a flexible HCT-CI using different cut off points to determine low-, intermediate-, and high-risk groups: the flexible HCT-CI Is the best predictor of NRM and OS in a population of patients undergoing allo-RIC. Biol Blood Marrow Transplant. 2010;16:413–20. 19922807 10.1016/j.bbmt.2009.11.008

[23] Backhaus D, Brauer D, Pointner R, Bischof L, Vucinic V, Franke GN, et al. A high hematopoietic cell transplantation comorbidity Index (HCT-CI) does not impair outcomes after non-myeloablative allogeneic stem cell transplantation in acute myeloid leukemia patients 60 years or older. Bone Marrow Transplant. 2023;58:30–8. 36195769 10.1038/s41409-022-01833-0 PMC9812784

[24] Fein JA, Shimoni A, Labopin M, Shem-Tov N, Yerushalmi R, Magen H, et al. The impact of individual comorbidities on non-relapse mortality following allogeneic hematopoietic stem cell transplantation. Leukemia. 2018;32:1787–94. 29950692 10.1038/s41375-018-0185-y

[25] Terwey TH, Hemmati PG, Martus P, Dietz E, Vuong LG, Massenkeil G, et al. A modified EBMT risk score and the hematopoietic cell transplantation-specific comorbidity index for pre-transplant risk assessment in adult acute lymphoblastic leukemia. Haematologica. 2010;95:810–8. 20007143 10.3324/haematol.2009.011809 PMC2864388

[26] Sorror ML, Logan BR, Zhu X, Rizzo JD, Cooke KR, McCarthy PL, et al. Prospective validation of the predictive power of the hematopoietic cell transplantation comorbidity index: a center for international blood and marrow transplant research study. Biol Blood Marrow Transplant. 2015;21:1479–87. 25862591 10.1016/j.bbmt.2015.04.004 PMC4512746

[27] Kröger N, Iacobelli S, Franke GN, Platzbecker U, Uddin R, Hübel K, et al. Dose-reduced versus standard conditioning followed by allogeneic stem-cell transplantation for patients with myelodysplastic syndrome: a prospective randomized phase III study of the EBMT (RICMAC Trial). J Clin Oncol. 2017;35:2157–64. 28463633 10.1200/JCO.2016.70.7349

[28] Penack O, Peczynski C, Mohty M, Yakoub-Agha I, Styczynski J, Montoto S, et al. How much has allogeneic stem cell transplant-related mortality improved since the 1980s? A retrospective analysis from the EBMT. Blood Adv. 2020;4:6283–90. 33351121 10.1182/bloodadvances.2020003418 PMC7756984

[29] Styczyński J, Tridello G, Koster L, Iacobelli S, van Biezen A, van der Werf S, et al. Death after hematopoietic stem cell transplantation: changes over calendar year time, infections and associated factors. Bone Marrow Transplant. 2020;55:126–36. 31455899 10.1038/s41409-019-0624-z PMC6957465

[30] DeFor TE, Majhail NS, Weisdorf DJ, Brunstein CG, McAvoy S, Arora M, et al. A modified comorbidity index for hematopoietic cell transplantation. Bone Marrow Transplant. 2010;45:933–8. 19802025 10.1038/bmt.2009.275

[31] Extermann M. Interaction between comorbidity and cancer. Cancer Control. 2007;14:13–22. 17242667 10.1177/107327480701400103

[32] Chiu BC, Gapstur SM, Greenland P, Wang R, Dyer A. Body mass index, abnormal glucose metabolism, and mortality from hematopoietic cancer. Cancer Epidemiol Biomark Prev. 2006;15:2348–54. 10.1158/1055-9965.EPI-06-0007 17164355

[33] Calle EE, Rodriguez C, Walker-Thurmond K, Thun MJ. Overweight, obesity, and mortality from cancer in a prospectively studied cohort of U.S. adults. N Engl J Med. 2003;348:1625–38. 12711737 10.1056/NEJMoa021423

[34] Sorror ML, Sandmaier BM, Storer BE, Maris MB, Baron F, Maloney DG, et al. Comorbidity and disease status based risk stratification of outcomes among patients with acute myeloid leukemia or myelodysplasia receiving allogeneic hematopoietic cell transplantation. J Clin Oncol. 2007;25:4246–54. 17724349 10.1200/JCO.2006.09.7865

[35] Mo XD, Xu LP, Liu DH, Zhang XH, Chen H, Chen YH, et al. The hematopoietic cell transplantation-specific comorbidity index (HCT-CI) is an outcome predictor for partially matched related donor transplantation. Am J Hematol. 2013;88:497–502. 23536204 10.1002/ajh.23443

[36] Michelis FV, Messner HA, Atenafu EG, McGillis L, Lambie A, Uhm J, et al. Patient age, remission status and HCT-CI in a combined score are prognostic for patients with AML undergoing allogeneic hematopoietic cell transplantation in CR1 and CR2. Bone Marrow Transplant. 2015;50:1405–10. 26168067 10.1038/bmt.2015.165

[37] Barba P, Martino R, Pérez-Simón JA, Fernández-Avilés F, Castillo N, Piñana JL, et al. Combination of the Hematopoietic Cell Transplantation Comorbidity Index and the European Group for Blood and Marrow Transplantation score allows a better stratification of high-risk patients undergoing reduced-toxicity allogeneic hematopoietic cell transplantation. Biol Blood Marrow Transplant. 2014;20:66–72. 24141006 10.1016/j.bbmt.2013.10.011

[38] Fattinger N, Roth JA, Baldomero H, Stolz D, Medinger M, Heim D, et al. External validation of the revised pretransplant assessment of mortality score in allogeneic hematopoietic cell transplantation: a cohort study. Hemasphere. 2022;6:e704. 35295589 10.1097/HS9.0000000000000704 PMC8920432

