# Supplementary material for: Influence of comorbidities on outcome in 1102 patients with an allogeneic hematopoietic stem cell transplantation
Source: Bone Marrow Transplant. 2024 Aug 13;59(11):1525–33. doi: 10.1038/s41409-024-02395-z (PMC11530370; doi:10.1038/s41409-024-02395-z)
Supplement: Supplementary file 1 — Supplementary_material [file 41409_2024_2395_MOESM1_ESM.doc]

**Supplementary Figure 1. Heat Map of the Correlation Matrix**


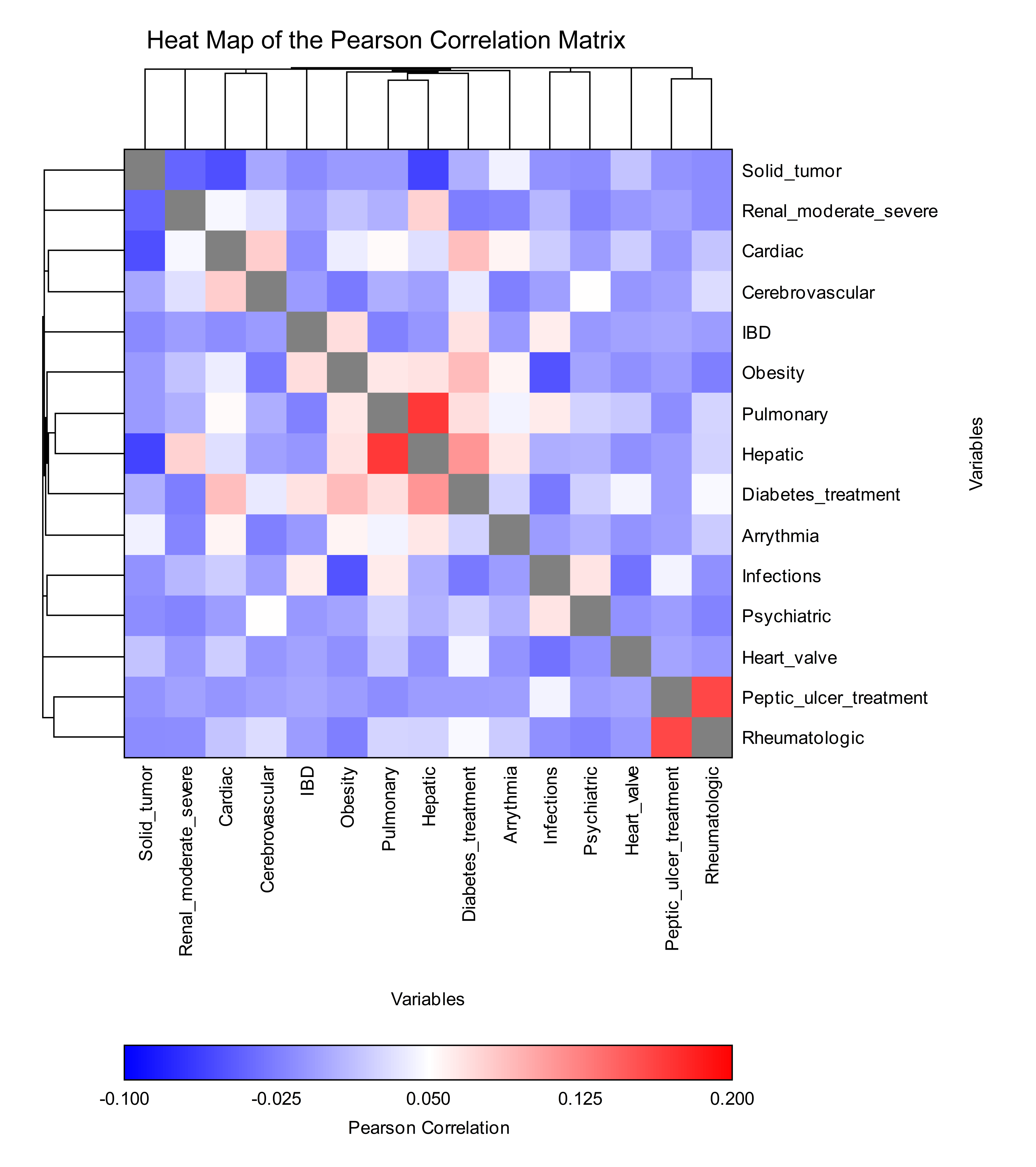


*Abbr: IBD* inflammatory bowel disease

The color red represents a positive association, pink and blush weaker positive correlations
